# EFFECT OF PHYSICAL EXERCISE ON THE HEALTH-RELATED QUALITY OF LIFE ASSESSED BY CLDQ OF LIVER CIRRHOSIS PATIENTS: SYSTEMATIC REVIEW WITH META-ANALYSIS

**DOI:** 10.1590/S0004-2803.24612025-024

**Published:** 2025-09-05

**Authors:** Fabiana Coelho Couto Rocha CORRÊA, Isabella Scarlatelli Telles Pires NADER, Laura Candeia Barbosa MUNIZ, Roberta Martins LOPES, Elirez Bezerra da SILVA

**Affiliations:** 1 Universidade do Estado do Rio de Janeiro, Rio de Janeiro, RJ, Brasil.; 2 Faculdade de Ciências Médicas e da Saúde de Juiz de Fora, Juiz de Fora, MG, Brasil.

**Keywords:** Exercise, quality of life, health-related quality of life, liver cirrhosis, Exercício, qualidade de vida, qualidade de vida relacionada à saúde, cirrose hepática

## Abstract

**Objective:**

To verify the effect of physical exercise on the quality of life of patients with liver cirrhosis (LC).

**Methods:**

the sample included controlled and randomized experimental studies of individuals with LC, at any stage of the disease, over 18 years of age, of both sexes, who performed any type of physical exercise compared to any other intervention or no intervention, with quality of life as the outcome assessed by the Chronic Liver Disease Questionnaire (CLDQ). The search for articles was conducted in 11 databases. The descriptors considered for the search were physical exercise, quality of life, liver cirrhosis, and their synonyms. The methodological quality and study bias were assessed using the Jadad scale and the RoB 2 scale, respectively. Review Manager 5.4 was used for the meta-analysis of the data. Quality of life was considered a continuous variable. The mean difference was considered as the effect measure. The analysis model was fixed-effect. The confidence level adopted was .05. The level of evidence for the meta-analysis results was assessed using the GRADE tool.

**Results:**

A meta-analysis of five studies, in which 153 participants with LC, of which 83 belonged to the physical exercise group and 70 to the control group, showed that the experimental group that performed physical exercise significantly increased quality of life by 0.46 [0.09 to 0.84]; *P*=.02. The level of evidence of the meta-analysis was considered high.

**Conclusion:**

Physical exercise led to an improvement in the health-related quality of life of patients with LC.

## HIGHLIGHTS

• Patients with liver cirrhosis (LC) have reduced health-related quality of life.

• However, the impact of reduced quality of life and the tools to improve quality of life in these patients are poorly studied.

• In this study, we explored the studies published in the scientific literature that investigated the effects of physical exercise on the quality of life of patients with LC.

• Only five studies were found in the scientific literature to be included in this meta-analysis.

• After analyzing these five studies, it was identified that physical exercise improves the health-related quality of life of patients with LC.

## RESUMO

**Objetivo:**

Verificar o efeito do exercício físico na qualidade de vida de pacientes com cirrose hepática (CH).

**Métodos:**

A amostra incluiu estudos experimentais controlados e randomizados de indivíduos com CH, em qualquer estágio da doença, maiores de 18 anos, de ambos os sexos, que realizaram qualquer tipo de exercício físico comparado a qualquer outra intervenção ou nenhuma intervenção, tendo como desfecho a qualidade de vida avaliada pelo questionário de doença crônica do fígado *Chronic Liver Disease Questionnaire* (CLDQ). A busca dos artigos foi realizada em 11 bases de dados. Os descritores considerados para a busca foram exercício físico, qualidade de vida, cirrose hepática e seus sinônimos. A qualidade metodológica e o viés do estudo foram avaliados por meio da escala de Jadad e da escala RoB 2, respectivamente. O Review Manager 5.4 foi utilizado para a metanálise dos dados. A qualidade de vida foi considerada uma variável contínua. A diferença média foi considerada como medida de efeito. O modelo de análise foi de efeito fixo. O nível de confiança adotado foi de 0,05. O nível de evidência dos resultados da metanálise foi avaliado por meio da ferramenta GRADE.

**Resultados:**

A metanálise de cinco estudos, que apresentou 153 participantes com CH, dos quais 83 pertenciam ao grupo de exercícios físicos e 70 ao grupo controle, mostrou que o grupo experimental que realizou exercícios físicos aumentou significativamente a qualidade de vida em 0,46 [0,09 a 0,84]; *P*=0,02. O nível de evidência da metanálise foi considerado alto.

**Conclusão:**

O exercício físico levou a uma melhora na qualidade de vida relacionada à saúde de pacientes com CH.

## INTRODUCTION

Liver cirrhosis (LC) is a highly prevalent disease, affecting approximately 112 million people worldwide^1^. It results from continuous and prolonged damage to the liver parenchyma cells, associated with diffuse regenerative nodules surrounded by fibrous tissue^2^. It is important to emphasize that patients with this condition experience a significant reduction in muscle mass, due to an imbalance between muscle formation and degradation, known as sarcopenia in patients with LC. In addition to its impact on mortality, sarcopenia also reduces the quality of life, functional capacity, and autonomy in patients with LC, as muscle weakness limits the ability to perform physical exercises and profoundly affects simple daily activities, contributing to fatigue. These effects can significantly impair the participation of patients with LC in the community, family life, and work performance^3^.

Quality of life is a broad concept that evaluates the patient’s life, encompassing various aspects such as physical health, social relationships, and socioeconomic, cultural, and emotional conditions. Health-related quality of life, on the other hand, refers to the health condition in the context of the patient’s pathology, providing a quantitative analysis of the consequences of a disease based on the individual’s self-perception of how it affects their useful life and vitality. It is the individual’s perception of their general health condition^4^.

Health-related quality of life can be assessed using various instruments, such as the Short Form 36 (SF-36), the World Health Organization Quality of Life 100 (WHOQOL-100), the abbreviated World Health Organization Quality of Life (WHOQOL-bref), the Total Quality of Work Life 42 (TQWL-42), and the Chronic Liver Disease Questionnaire (CLDQ), among others^5^. The CLDQ is a validated questionnaire for evaluating the quality of life in patients with chronic liver disease. This questionnaire consists of 29 items, distributed across 6 domains: fatigue (questions 2, 4, 8, 11, and 13), activity (questions 7, 9, and 14), emotional function (questions 10, 12, 15, 16, 19, 20, 24, and 26), abdominal symptoms (questions 1, 5, and 17), systemic symptoms (questions 3, 6, 21, 23, and 27), and worry (questions 18, 22, 25, 28, and 29). The scores for each domain range from 1 to 7. The score for each domain is obtained by summing the responses and dividing by the number of questions in that domain. The total CLDQ score is obtained by summing the domains and dividing by 6^6^.

Considering that liver cirrhosis (LC) reduces functional capacity and autonomy, as well as muscle strength and both respiratory and peripheral muscle endurance, factors that directly impact quality of life, it is crucial for both clinical practice and scientific research to investigate the potential benefits of physical exercise for these patients. Evidence suggests that physical exercise improves functional capacity, strength, and muscle endurance in individuals with LC^7^ ^-^ ^11^.

Aamann et al. (2018)^12^ conducted a meta-analysis to assess the beneficial effects of physical exercise compared to sham exercise or no exercise in individuals with cirrhosis. However, this meta-analysis has not been updated.

Given the severity and prevalence of LC, the associated pathophysiological changes, and the lack of recent meta-analyses on the impact of physical exercise on quality of life in these patients, the present study aimed to evaluate the effects of physical exercise on the quality of life of individuals with LC.

## METHODS

### Study design

Systematic review with meta-analysis written according to the Preferred Reporting Items for Systematic Reviews and Meta-Analyses (PRISMA)^13^, registered under number CRD42024540326 in PROSPERO.

### Inclusion criteria for studies

Inclusion criteria followed the population, intervention, comparison, outcome, and study design (PICOS) strategy^14^, as follows: controlled and randomized experimental studies with individuals with LC, at any stage of the disease, over 18 years of age, of any sex, who performed any type of physical exercise compared to any other intervention or no intervention, with the outcome being quality of life assessed by the Chronic Liver Disease Questionnaire (CLDQ).

### Search strategy and study selection

The search and selection were conducted from January to June 2024 by three researchers, with a fourth to resolve discrepancies, in the following databases: United States National Library of Medicine - Medical Publications (MEDLINE-PubMed), Virtual Health Library - Latin American and Caribbean Literature in Health Sciences (BVS-LILACS), Scientific Electronic Library Online (SciELO), Cochrane Central Register of Controlled Trials, Physiotherapy Evidence Database (PEDro), Web of Science, Scopus, SPORTDiscus, ScienceDirect, Excerpta Medical Database (EMBASE), and Cumulative Index to Nursing and Allied Health Literature (CINAHL). Descriptors for the search were determined according to the Health Science Descriptors (DeCS), Medical Subject Headings (MeSH), and the researchers’ experience. The Boolean operator AND was used between descriptors and OR between synonyms. Search phrases for each database are presented in APPENDIX 1.

**APPENDIX 1:** Search phrases for each database.

Database	Phrase	Number of Titles Recovered
BVS-LILACS	(exercise OR “physical activity” OR “aerobic exercise” OR “physical exercise” OR “acute exercise” OR “isometric exercise” OR “exercise training” OR “training” OR “resistance training” OR “strength training” OR “weight bearing strengthening program” OR “respiratory muscle training” OR “breathing exercises”) AND (“Quality of Life” OR HRQOL OR “Health-Related Quality Of Life” OR SF-36 OR CLDQ) AND (“liver cirrhosis” OR “hepatic cirrhosis” OR “liver fibrosis”)	0
	(exercise OR “physical activity” OR “aerobic exercise” OR “physical exercise” OR “acute exercise” OR “isometric exercise” OR “exercise training” OR “training” OR “resistance training” OR “strength training” OR “weight bearing strengthening program” OR “respiratory muscle training” OR “breathing exercises”) AND (“liver cirrhosis” OR “hepatic cirrhosis” OR “liver fibrosis”)	0
	(“Quality of Life” OR HRQOL OR “Health-Related Quality Of Life” OR SF-36 OR CLDQ) AND (“liver cirrhosis” OR “hepatic cirrhosis” OR “liver fibrosis”)	29
CINAHL	SU ( exercise OR “physical activity” OR “aerobic exercise” OR “physical exercise” OR “acute exercise” OR “isometric exercise” OR “exercise training” OR “training” OR “resistance training” OR “strength training” OR “weight bearing strengthening program” OR “respiratory muscle training” OR “breathing exercises” ) AND SU ( “Quality of Life” OR HRQOL OR “Health-Related Quality Of Life” OR SF-36 OR CLDQ ) AND SU ( “liver cirrhosis” OR “hepatic cirrhosis” OR “liver fibrosis” )	7
	SU ( exercise OR “physical activity” OR “aerobic exercise” OR “physical exercise” OR “acute exercise” OR “isometric exercise” OR “exercise training” OR “training” OR “resistance training” OR “strength training” OR “weight bearing strengthening program” OR “respiratory muscle training” OR “breathing exercises” ) AND SU ( “liver cirrhosis” OR “hepatic cirrhosis” OR “liver fibrosis” )	38
	SU ( “Quality of Life” OR HRQOL OR “Health-Related Quality Of Life” OR SF-36 OR CLDQ ) AND SU (“liver cirrhosis” OR “hepatic cirrhosis” OR “liver fibrosis” )	121
Cochrane	“liver cirrhosis” OR “hepatic cirrhosis” OR “liver fibrosis” in Title Abstract Keyword AND “Quality of Life” OR HRQOL OR “Health-Related Quality Of Life” OR SF-36 OR CLDQ in Title Abstract Keyword AND exercise OR “physical activity” OR “aerobic exercise” OR “physical exercise” OR “acute exercise” OR “isometric exercise” OR “exercise training” OR “training” OR “resistance training” OR “strength training” OR “weight bearing strengthening program” OR “respiratory muscle training” OR “breathing exercises” in Title Abstract Keyword	82
	“liver cirrhosis” OR “hepatic cirrhosis” OR “liver fibrosis” in Title Abstract Keyword AND “Quality of Life” OR HRQOL OR “Health-Related Quality Of Life” OR SF-36 OR CLDQ in Title Abstract Keyword	669
	“liver cirrhosis” OR “hepatic cirrhosis” OR “liver fibrosis” in Title Abstract Keyword AND exercise OR “physical activity” OR “aerobic exercise” OR “physical exercise” OR “acute exercise” OR “isometric exercise” OR “exercise training” OR “training” OR “resistance training” OR “strength training” OR “weight bearing strengthening program” OR “respiratory muscle training” OR “breathing exercises” in Title Abstract Keyword	422
EMBASE	(‘exercise’/exp OR exercise OR ’physical activity’/exp OR ’physical activity’ OR ’aerobic exercise’/exp OR ’aerobic exercise’ OR ’physical exercise’/exp OR ’physical exercise’ OR ’acute exercise’/exp OR ’acute exercise’ OR ’isometric exercise’/exp OR ’isometric exercise’ OR ’exercise training’/exp OR ’exercise training’ OR ’training’/exp OR ’training’ OR ’resistance training’/exp OR ’resistance training’ OR ’strength training’/exp OR ’strength training’ OR ’weight bearing strengthening program’ OR ’respiratory muscle training’/exp OR ’respiratory muscle training’ OR ’breathing exercises’/exp OR ’breathing exercises’) AND (‘quality of life’:ti,ab,kw OR hrqol:ti,ab,kw OR ’health related quality of life’:ti,ab,kw OR ’sf 36’:ti,ab,kw OR cldq:ti,ab,kw) AND (‘liver cirrhosis’:ti,ab,kw OR ’hepatic cirrhosis’:ti,ab,kw OR ’liver fibrosis’:ti,ab,kw)	106
	(exercise:ti,ab,kw OR ’physical activity’:ti,ab,kw OR ’aerobic exercise’:ti,ab,kw OR ’physical exercise’:ti,ab,kw OR ’acute exercise’:ti,ab,kw OR ’isometric exercise’:ti,ab,kw OR ’exercise training’:ti,ab,kw OR ’training’:ti,ab,kw OR ’resistance training’:ti,ab,kw OR ’strength training’:ti,ab,kw OR ’weight bearing strengthening program’:ti,ab,kw OR ’respiratory muscle training’:ti,ab,kw OR ’breathing exercises’:ti,ab,kw) AND (‘liver cirrhosis’:ti,ab,kw OR ’hepatic cirrhosis’:ti,ab,kw OR ’liver fibrosis’:ti,ab,kw) AND ’article’/it	655
	(‘liver cirrhosis’:ti,ab,kw OR ’hepatic cirrhosis’:ti,ab,kw OR ’liver fibrosis’:ti,ab,kw) AND (‘quality of life’:ti,ab,kw OR hrqol:ti,ab,kw OR ’health-related quality of life’:ti,ab,kw OR ’sf 36’:ti,ab,kw OR cldq:ti,ab,kw) AND ’article’/it	610
MEDLINE-Pubmed	((((“liver cirrhosis”[Title/Abstract]) OR (“hepatic cirrhosis”[Title/Abstract])) OR (“liver fibrosis”[Title/Abstract])) AND (((((“Quality of Life”[Title/Abstract]) OR (HRQOL[Title/Abstract])) OR (“Health Related Quality Of Life”[Title/Abstract])) OR (SF-36[Title/Abstract])) OR (CLDQ[Title/Abstract]))) AND (((((((((((((exercise[Title/Abstract]) OR (“physical activity”[Title/Abstract])) OR (“aerobic exercise”[Title/Abstract])) OR (“physical exercise”[Title/Abstract])) OR (“acute exercise”[Title/Abstract])) OR (“isometric exercise”[Title/Abstract])) OR (“exercise training”[Title/Abstract])) OR (“training”[Title/Abstract])) OR (“resistance training”[Title/Abstract])) OR (“strength training”[Title/Abstract])) OR (“weight bearing strengthening program”[Title/Abstract])) OR (“respiratory muscle training”[Title/Abstract])) OR (“breathing exercises”[Title/Abstract]))	24
MEDLINE-Pubmed	(((“liver cirrhosis”[Title/Abstract]) OR (“hepatic cirrhosis”[Title/Abstract])) OR (“liver fibrosis”[Title/Abstract])) AND (((((“Quality of Life”[Title/Abstract]) OR (HRQOL[Title/Abstract])) OR (“Health Related Quality Of Life”[Title/Abstract])) OR (SF-36[Title/Abstract])) OR (CLDQ[Title/Abstract]))	540
	(((“liver cirrhosis”[Title/Abstract]) OR (“hepatic cirrhosis”[Title/Abstract])) OR (“liver fibrosis”[Title/Abstract])) AND (((((((((((((exercise[Title/Abstract]) OR (“physical activity”[Title/Abstract])) OR (“aerobic exercise”[Title/Abstract])) OR (“physical exercise”[Title/Abstract])) OR (“acute exercise”[Title/Abstract])) OR (“isometric exercise”[Title/Abstract])) OR (“exercise training”[Title/Abstract])) OR (“training”[Title/Abstract])) OR (“resistance training”[Title/Abstract])) OR (“strength training”[Title/Abstract])) OR (“weight bearing strengthening program”[Title/Abstract])) OR (“respiratory muscle training”[Title/Abstract])) OR (“breathing exercises”[Title/Abstract]))	595
PEDro	“liver cirrhosis”	8
SciELO	(exercise OR “physical activity” OR “aerobic exercise” OR “physical exercise” OR “acute exercise” OR “isometric exercise” OR “exercise training” OR “training” OR “resistance training” OR “strength training” OR “weight bearing strengthening program” OR “respiratory muscle training” OR “breathing exercises”) AND (“Quality of Life” OR HRQOL OR “Health-Related Quality Of Life” OR SF-36 OR CLDQ) AND (“liver cirrhosis” OR “hepatic cirrhosis” OR “liver fibrosis”) AND (“Quality of Life” )	0
	(exercise OR “physical activity” OR “aerobic exercise” OR “physical exercise” OR “acute exercise” OR “isometric exercise” OR “exercise training” OR “training” OR “resistance training” OR “strength training” OR “weight bearing strengthening program” OR “respiratory muscle training” OR “breathing exercises”) AND (“liver cirrhosis” OR “hepatic cirrhosis” OR “liver fibrosis”) AND (“Quality of Life” )	0
	(“Quality of Life” OR HRQOL OR “Health-Related Quality Of Life” OR SF-36 OR CLDQ) AND (“liver cirrhosis” OR “hepatic cirrhosis” OR “liver fibrosis”) AND (“Quality of Life”)	0
ScienceDirect	(“liver cirrhosis”) AND (“Quality of Life” OR HRQOL OR “Health-Related Quality Of Life” OR SF-36 OR CLDQ) AND (exercise OR “physical activity” OR training)	9
	(“liver cirrhosis”) AND (exercise OR “physical activity” OR training)	83
Scopus	( TITLE-ABS-KEY ( exercise OR ”physical activity” OR ”aerobic exercise” OR ”physical exercise” OR ”acute exercise” OR ”isometric exercise” OR ”exercise training” OR ”training” OR ”resistance training” OR ”strength training” OR ”weight bearing strengthening program” OR ”respiratory muscle training” OR ”breathing exercises” ) AND TITLE-ABS-KEY (”Quality of Life” OR hrqol OR ”Health-Related Quality Of Life” OR sf-36 OR cldq ) AND TITLE-ABS-KEY (”liver cirrhosis” OR ”hepatic cirrhosis” OR ”liver fibrosis” ) )	334
	( TITLE-ABS-KEY ( exercise OR ”physical activity” OR ”aerobic exercise” OR ”physical exercise” OR ”acute exercise” OR ”isometric exercise” OR ”exercise training” OR ”training” OR ”resistance training” OR ”strength training” OR ”weight bearing strengthening program” OR ”respiratory muscle training” OR ”breathing exercises” ) AND TITLE-ABS-KEY ( ”liver cirrhosis” OR ”hepatic cirrhosis” OR ”liver fibrosis” ) ) AND ( LIMIT-TO ( DOCTYPE , ”ar” ) )	2439
	( TITLE-ABS-KEY ( ”Quality of Life” OR hrqol OR ”Health-Related Quality Of Life” OR sf-36 OR cldq) AND TITLE-ABS-KEY ( ”liver cirrhosis” OR ”hepatic cirrhosis” OR ”liver fibrosis” ) ) AND ( LIMIT-TO ( DOCTYPE , ”ar” ) )	2383
SPORTDiscus	SU ( exercise OR “physical activity” OR “aerobic exercise” OR “physical exercise” OR “acute exercise” OR “isometric exercise” OR “exercise training” OR “training” OR “resistance training” OR “strength training” OR “weight bearing strengthening program” OR “respiratory muscle training” OR “breathing exercises” ) AND SU ( “Quality of Life” OR HRQOL OR “Health-Related Quality Of Life” OR SF-36 OR CLDQ ) AND SU ( “liver cirrhosis” OR “hepatic cirrhosis” OR “liver fibrosis” )	0
	SU ( exercise OR “physical activity” OR “aerobic exercise” OR “physical exercise” OR “acute exercise” OR “isometric exercise” OR “exercise training” OR “training” OR “resistance training” OR “strength training” OR “weight bearing strengthening program” OR “respiratory muscle training” OR “breathing exercises” ) AND SU ( “liver cirrhosis” OR “hepatic cirrhosis” OR “liver fibrosis” )	1
	SU ( “Quality of Life” OR HRQOL OR “Health-Related Quality Of Life” OR SF-36 OR CLDQ ) AND SU ( “liver cirrhosis” OR “hepatic cirrhosis” OR “liver fibrosis” )	0
Web of Science	exercise OR “physical activity” OR “aerobic exercise” OR “physical exercise” OR “acute exercise” OR “isometric exercise” OR “exercise training” OR “training” OR “resistance training” OR “strength training” OR “weight bearing strengthening program” OR “respiratory muscle training” OR “breathing exercises” (Topic) AND “Quality of Life” OR HRQOL OR “Health-Related Quality Of Life” OR SF-36 OR CLDQ (Topic) AND “liver cirrhosis” OR “hepatic cirrhosis” OR “liver fibrosis” (Topic)	52
	exercise OR “physical activity” OR “aerobic exercise” OR “physical exercise” OR “acute exercise” OR “isometric exercise” OR “exercise training” OR “training” OR “resistance training” OR “strength training” OR “weight bearing strengthening program” OR “respiratory muscle training” OR “breathing exercises” (Topic) AND “Quality of Life” OR HRQOL OR “Health-Related Quality Of Life” OR SF-36 OR CLDQ (Topic) AND “liver cirrhosis” OR “hepatic cirrhosis” OR “liver fibrosis” (Topic)	953
	“Quality of Life” OR HRQOL OR “Health-Related Quality Of Life” OR SF-36 OR CLDQ (Topic) AND “liver cirrhosis” OR “hepatic cirrhosis” OR “liver fibrosis” (Topic)	1000
**Total**		**11160**

To search for scientific articles, three combinations of descriptors were used for each database: (1) liver cirrhosis, physical exercise, and quality of life; (2) liver cirrhosis and physical exercise; and (3) liver cirrhosis and quality of life. Synonyms for the three descriptors were considered in the three combinations used.

Study selection was performed in three stages: (1) exclusion of duplicate studies from the databases using the application EndNote; (2) exclusion of studies whose titles and abstracts did not meet the inclusion criteria; and (3) exclusion of studies whose full texts did not meet the inclusion criteria.

### Assessment of methodological quality and risk of bias of the studies

To assess the methodological quality of the studies, the Jadad scale was used, applied by three independent, qualified researchers, with a fourth to resolve discrepancies. The following methodological criteria were considered: 1a) the study was described as randomized; 1b) the randomization was performed correctly; 2a) it was a double-blind trial; 2b) blinding was adequately conducted; and 3) description of sample loss. If items 1a, 2a, and 3 were met, the study earned 1 point per item. If items 1b and 2b were met, the study earned an additional point per item. Additionally, non-fulfillment of items 1b and 2b resulted in a 1-point deduction from the study’s score for items 1a and 2a, respectively.

The RoB 2 scale was used to assess the risk of bias in studies, applied by three independent, qualified researchers, with a fourth to resolve discrepancies. The RoB 2 scale is recommended by the Cochrane Collaboration and involves using the Cochrane Risk of Bias Tool version 2 (RoB 2), which assesses six criteria: randomization, deviations from intended interventions, missing outcome data, outcome measurement, reported result selection, and other biases^15^.

### Data extraction

From the selected studies, the following data were extracted by three independent researchers, with a fourth to resolve discrepancies: participant profile, sample size, intervention protocol, and quality of life outcome with their respective significance levels. In cases of missing data, the authors of the selected studies were contacted via email.

### Data analysis

The RevMan5.4 program, available for free at http://community.cochrane.org/tools/review-production-tools/revman-5.4, was used for meta-analysis. Quality of life was considered a continuous variable. The mean difference was considered as the effect measure. The fixed-effect analysis model was used due to low inconsistency. A 95% confidence interval (95%CI) was used for results from each study and the meta-analysis. The confidence level adopted was .05. Publication bias was assessed using a Funnel Plot and confirmed by the Egger test from StatsDirect version 3.

### Level of evidence of the meta-analysis

The level of evidence of the meta-analysis result was assessed using the grading of recommendations assessment development and evaluation (GRADE) tool, by three independent researchers with a fourth to resolve discrepancies. In the GRADE tool, randomized controlled trials start with a high level of evidence, and during the assessment, the quality of studies may be reduced due to five factors: methodological limitations, inconsistency, indirect evidence, imprecision, and publication bias. At the end of the analysis, the GRADE system evaluates the level of evidence of the meta-analysis as high, moderate, low, or very low^16^.

## RESULTS

**FIGURE 1:**
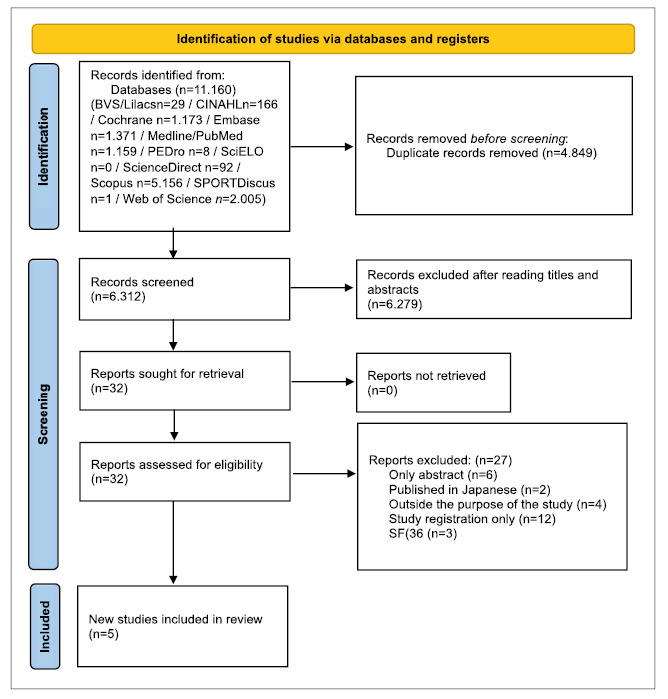
Flow diagram of controlled and randomized clinical trials included in the meta-analysis.

The number of studies retrieved and selected and the reasons for exclusions (Figure 1).

All patients had a diagnosis of CL and were randomized and allocated into two groups. In both groups, there was a similar age (mean) of 57.3 years, with the exercise group (EG) comprising 57% males and the control group (CG) comprising 66.7% males (Table 1). Aerobic exercises on a cycle ergometer were performed for eight to 12 weeks, two to four times per week, at an intensity of 60% to 80% VO2peak, maximum heart rate (HRmax), or reserve heart rate (HRreserve), with a duration of 30 to 60 minutes. Strength training exercises were performed over 12 weeks, three times per week, with a duration of 30 minutes. Activities of daily living exercises were performed using elastic bands, with resistance increased when the individual could perform 10 repetitions of each movement pattern without significant fatigue or loss of proper execution (Table 2).

**TABLE 1:** Characteristics of the participants.

RCT/Year	Sample size, age (years), and sex (M/F)	Child-Turcotte-Pugh Classification (Severity of LC)	MELD Score (Mean ± *SD*)	Etiology of the LC	Signs and symptoms (yes/no)
**Kruger et al., 2018** ^23^	Exercise group: n=20^a^,	Child classification A	Exercise group: 9.05^b^	Alcoholic	Not informed by the authors
	age 53.0±8.3,		Control group: 9.7^b^	Hepatitis C	
	sex 10/10			NASH	
	Control group: n=20,			Other	
	age 56.4±8.5, sex 13/7				
**Lai et al., 2021** ^24^	Exercise group: n=58^c^,	Child classification B/C	Exercise group: 16.67d±19.77^d^	Alcoholic	Presence of Hepatic y Encephalopath (EG:12/46; CG:6/19)
	age 61.33d±7.6d, sex 29/29		Control group: 14^d^±13.36^d^	NASH	
	Control group: n=25^e^,			Hepatitis C	Presence of ascites (EG:11/47; CG:9/16)
	age 58.33d±23.58d, sex 18/7			Autoimmune	
				Other	
**Macías-Rodrigues et al., 2016** ^25^	Exercise Group: n=13,	Child class A/B	Exercise group: 9	Hepatitis C	Presence of Hepatic Encephalopathy (EG:3/10; CG:3/9)
	age 53 (48-55), sex 9/4.		Control group: 12	NASH	Presence of ascites (EG:0/12; CG: 2/10)
	Control Group: n=12,		SD not reported by authors	Alcoholic	History of variceal bleeding (EG:4/9; CG:3/9)
	age 51 (38-57), sex 10/2.			Other	Presence of small varices (EG:8/5; CG:9/3)
**Sirisunhirun et al., 2022** ^26^	Exercise group: n=20,	Child classification A	Exercise group: 7.95±1.5	Alcoholic	Not informed by the authors
	age 55.6±8.9, sex 15/5		Control group: 7.95±1.3	Hepatitis B	
	Control group: n=20,			Hepatitis C	
	age 57.1±6.7, sex 11/9			NASH	
**Zenith et al., 2014** ^27^	Exercise group: n=9,	Child classification A/B	Exercise group: 9.7±2.4	Alcoholic	Presence of ascites (EG:0/9; CG: 0/10)
	age 56.4±7.7, sex 7/2		Control group: 10.2±1.9	Hepatitis B	
	Control group: n=10,			Hepatitis C	History of varices (EG:8/1; CG:10/0)
	age 58.6±5.8, sex 8/2			NASH	
				Other	

RCT: randomized controlled trial; LC: liver cirrhosis; M: male; F: female; MELD: Model for End-stage Liver Disease; SD: standard deviation; EG: exercise group; CG: control group; NASH: nonalcoholic steatohepatitis.

^a^Initially, there were 20 volunteers randomized in the EG; however, only 11 volunteers completed the protocol, as shown in the Forest Plot; ^b^Standard deviation not reported by the author; ^c^Initially, there were 58 volunteers randomized in the EG; however, only 43 volunteers completed the protocol, as shown in the Forest Plot; ^d^The study author presented the values as median and interquartile range, which were converted to mean and standard deviation; ^e^Initially, there were 25 volunteers randomized in the CG; however, only 20 volunteers completed the protocol, as shown in the Forest Plot.

**TABLE 2:** Characteristics of the physical exercises performed and quality of life outcomes.

RCT/Year	Duration / Weekly Frequency / Length of the session	Intervention protocol	Quality of life (CLDQ) result	Exercise group	Control group	Between groups
**Kruger et al., 2018** ^23^	8 weeks / 3-2 days / 30-60 minutes	Exercise group: home exercises - Cycle ergometer / 60-80% HR reserve or 14 to 15 points on the Borg scale / 5 minutes of warm-up on the cycle ergometer / 20 to 50 minutes on the cycle ergometer depending on tolerance / 5 minutes of cool-down on the cycle ergometer.	CLDQ Total	Pre 5.06±1.09	Pre 5.22±1.36	*P=*.57
		Control group: advised to maintain regular activities during the study.		Post 4.76±1.16	Post 5.03±1.34	
				(*P=*.13)	(*P=*.29)	
			Abdominal symptoms	Post 5.43±1.54	Pre 4.93±1.82	*P=*.49
				Pre 5.30±1.43	Post 5.32±1.71	
				(*P=*.66)	(*P=*.06)	
			Systemic symptoms	Pre 5.01±1.12	Pre 5.04±1.51	*P=*.07
				Post 4.53±1.36	Post 5.10±1.49	
				(*P=*.07)	(*P=*.72)	
			Activity	Pre 5.08±1.42	Pre 5.32±1.71	*P=*.80
				Post 4.73±1.50	Post 4.77±2.13	
				(*P=*.20)	(*P=*.21)	
			Emotional function	Pre 5.31±1.04	Pre 5.63±1.32	*P=*.16
				Post 4.84±1.22	Post 5.46±1.33	
				(*P=*.03)	(*P=*.23)	
			Worry	Pre 5.30±1.33	Pre 5.42±1.69	*P=*.83
				Post 5.18±1.37	Post 5.34±1.75	
				(*P=*.59)	(*P=*.83)	
			Fatigue	Pre 4.28±1.53	Pre 4.46±1.52	*P=*.72
				Post 4.14±1.35	Post 4.16±1.68	
				(*P=*.55)	(*P=*.24)	
**Lai et al., 2021** ^24^	12 weeks / 3 days / 30 minutes.	Exercise group: home exercise program using video lessons from the “Strong-for-Life” program, consisting of 11 exercises related to daily living activities. Elastic bands of varying thicknesses, coded by color, were used to individualize resistance. The exercises included diagonal and rotational movement patterns, as well as movements associated with functional activities. Exercises were performed either seated or standing. Participants were instructed to increase the resistance when they could perform 10 repetitions of each movement pattern without significant fatigue or loss of proper execution. The program included 5 minutes of warm-up, 25 minutes of strengthening, and 5 minutes of cool-down exercises. Each week, volunteers were instructed to increase their step count by 500 steps compared to the previous week.	CLDQ Total:	Pre 4.6±1.84	Pre 4.83±.24	*P=*.09
		Control group: received written recommendations on the importance of physical exercise.		Post 4.97±1.61	Post 4.33±1.76	
				(*P=*not reported by the authors)	(*P=*not reported by the authors)	
**Macías-Rodrigues et al., 2016** ^25^	14 weeks/3 days/40 minutes	Exercise group: nutritional therapy (65% of carbohydrates, 1.2 g/kg weight per day of protein, and the rest from lipids) + cycle ergometer/12-14 Borg’s perceived exertion rating scale, 60-80% HR_max_ age-adjusted. Each session had three phases:	CLDQ	Pre 4.0±0.9	Pre 5.5±0.48	*P* value between groups not reported by the authors
		warm up, main phase and cool down (10, 20 and 10 min, respectively) + kinesiotherapy 30 min of activities aimed at improving muscle strength and elasticity, coordination and balance.	Total:	Post 5.8 0.5	Post 5.7±0.38	
		Control group: nutritional therapy (65% of carbohydrates, 1.2 g/kg weight per day of protein, and the rest from lipids)		(*P=*0.182)	(*P=*0.475)	
**Sirisunhirun et al., 2022** ^26^	12 weeks / 4 days / 40 minutes	Exercise group: 60 to 80% of max HR	CLDQ	Pre 5.6±0.9	Pre 5.1±1.1	*P=*.23
		Control group: received general exercise counseling and were encouraged to continue their routine daily activities	Total	Post 5.9±0.8	Post 5.3±1.1	
		Included 5 minutes of warm-up, isotonic exercises in 2 sets/15 repetitions (squats, lunges, sumo squats, bench press, trunk extension, overhead press, frontal and lateral shoulder raises, biceps curl, and trunk twists) for 30 minutes, and 5 minutes of cool-down.		(*P=*.03)	(*P=*.01)	
		.	Abdominal symptoms	Pre 5.9±1.0	Pre 5.3±1.3	*P=*.62
				Post 5.9±1.3	Post 5.3±1.4	
				(*P=*.95)	(*P=*1.0)	
			Fatigue	Pre 5.1±1.1	Pre 4.7±1.1	*P=*.5
				Post 5.6±0.9	Post 4.9±1.1	
				(*P=*.009)	(*P=*.02)	
			Systemic symptoms	Pre 5.8±0.8	Pre 5.2±1.2	*P=*.12
				Post 6.0±0.7	Post 4.7±0.8	
				(*P=*.22)	(*P=*.95)	
			Activity	Pre5.8±0.8	Pre 5.3±1.2	*P*= .08
				Post 6.3±0.9	Post 6.3±1.0	
				(*P=*.01)	(*P=*.13)	
			Emotional function	Pre 5.4±1.0	Pre 4.9 ± 1.3	*P=*.68
				Post 5.8±0.9	Post 5.8±0.9	
				(*P=*.03)	(*P=*.03)	
			Worry	Pre:5.8±1.5	Pre 5.1±1.4	*P=*.72
				Post: 5.9±1.1	Post 5.4±1.4	
				(*P=*.66)	(*P=*.06)	
**Zenith et al., 2014** ^27^	8 weeks / 3 days / 40-60 minutes	Exercise group: cycle ergometer / 60-80% VO_2peak_ / 5-minute warm-up; Start with 30 minutes per session, with an increase of 2.5 minutes per session each week, 5-minute cool-down..	CLDQ Total	Pre 5.72±0.63	Pre 5.42±0.81	*P=*.15
				Post 6.01±0.63	Post 5.39±0.96	
				(*P=*.19)	(*P=*.75)	
		Control group: instructed to maintain regular activities throughout the study	Abdominal symptoms	Pre 6.44±0.76	Pre 6.16±0.91	*P=*.71
				Post 6.48±1.03	Post 6.10±1.0	
				(*P=*.81)	(*P=*.75)	
			Systemic symptoms	Pre 5.96±0.91	Pre 5.86±0.53	*P=*.24
				Post 6.00±1.01	Post 5.6±0.87	
				(*P=*.77)	(*P=*.19)	
			Activity	Pre 5.52±1.36	Pre 6.07±0.63	*P=*.01
				Post 6.41±0.62	Post 5.33±1.12	
				(*P=*.08)	(*P=*.03)	
			Emotional ‘function	Pre 5.81±0.5	Pre 5.5±1.04	*P=*.50
				Post 5.89±0.82	Post 5.47±0.89	
				(*P=*.73)	(p = .75)	
			Worry	Pre 6.16±1.00	Pre 5.48±1.12	*P=*.46
				Post 6.11±1.44	Post 5.0±1.74	
				(*P=*.91)	(*P=*.28)	
			Fatigue	Pre 4.64±1.52	Pre 4.88 ± 1.12	*P=*.01
				Post 5.62±0.71	Post 4.93±0.93	
				(*P=*.03)	(*P=*.84)	

All studies received a score of 3 on the Jadad Scale used to assess the methodological quality of the RCTs, with the absence of double-blinding being common to the five studies included (Table 3).

**TABLE 3:** Methodological quality of the randomized controlled trials assessed by the Jadad Scale.

Authors	Criteria					
	Is randomization mentioned?	Is the randomization appropriate?	Is blinding mentioned?	Is the blinding method appropriate?	Are the losses and exclusions described?	Points
Kruger et al. (2018)^23^	1	1	0	0	1	3
Lai et al. (2021)^24^	1	1	0	0	1	3
Mácias-Rodriguez et al. (2016)^25^	1	1	0	0	1	3
Sirisunhirun et al. (2022)^26^	1	1	0	0	1	3
Zenith et al. (2014)^27^	1	1	0	0	1	3

**FIGURE 2:**
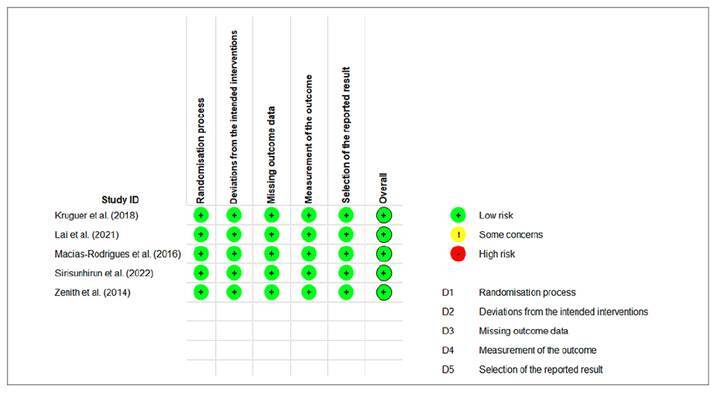
Results of risk of bias assessment of the randomized controlled trials assessed by the RoB 2 Cochrane Collaboration Tool^14^.

All studies demonstrated a low risk of bias according to the Cochrane RoB 2 tool used to assess the risk of bias in the RCTs (Figure 2).

**FIGURE 3:**
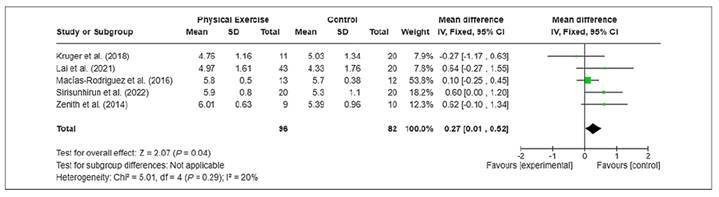
Forest plot of the total quality of life results measured by the CLDQ questionnaire.

The Forest plot of the meta-analysis (Figure 3).

**FIGURE 4:**
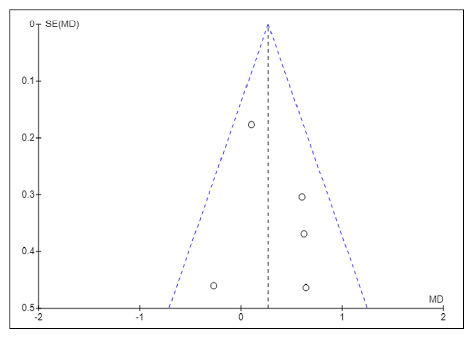
Funnel Plot: the X-axis shows the mean differences in quality of life between the experimental and control groups; the Y-axis shows the standard error of these differences. The small circles represent the studies plotted by mean difference and standard error. The Egger test result of -0.943; *P*=.35 confirmed the Funnel Plot result: no suspicion of publication bias.

The Funnel Plot subjectively indicated that there was no suspicion of publication bias in the studies evaluating the effectiveness of physical exercise on the quality of life in patients with LC. The Egger test result confirmed the Funnel Plot result: no suspicion of publication bias (Figure 4).

The level of evidence for the meta-analysis, assessed using the Grading of Recommendations Assessment, Development, and Evaluation (GRADE) tool, was considered high (Table 4).

**TABLE 4:** Level of evidence for the meta-analysis, assessed using the Grading of Recommendations Assessment, Development and Evaluation (GRADE) tool.

-Certainty assessment							Nº of patients		Effect		Certainty	Importance
Nº of studies	Study design	Risk of bias	Inconsistency	Indirect evidence	Imprecision	Other considerations	Physical exercise	Control	Relative (95% CI)	Absolut (95% CI)		
**Quality of Life (assessed by: CLDQ)**												
5	Controlled and rando­mized trials	Not serious^a^	Not serious^b^	Not serious^c^	Not serious^d^	None^e^	96	82	-	mean 0.27 (0.01 lower to 0.52 higher)	🜨 🜨 🜨 🜨 High	

CI: confidence interval. ^a^All studies evaluated by the RoB-2 had a low risk of bias; ^b^I2=20%, P=0.29; ^c^The included studies met the inclusion criteria, which were consistent with the aim of the metaanalysis; ^d^N=178 participants; ^e^There was no publication bias, as demonstrated by the subjective analysis of the Funnel Plot and objective analysis of the Egger test.

## DISCUSSION

The objective of this meta-analysis was to evaluate the effect of physical exercise on the quality of life of patients with LC. The results indicated that the practice of physical exercise, whether aerobic and/or muscular strength, promoted an improvement of 0.27 points [0.01 to 0.52]; *P*=0.04 in the quality of life of these patients (Figure 3). This improvement in the quality of life of these patients can be attributed to the regular practice of physical exercise, which contributed to improving functional capacity, muscular strength and muscular endurance, both respiratory and peripheral, which are factors that negatively impact the quality of life of patients with LC^7^ ^-^ ^11^.

According to Mouelhi et al. (2020)^17^, an increase of 0.27 points in favor of the group that performed physical exercises can be considered a clinically significant difference in improving health-related quality of life. This reinforces the importance of practicing physical exercises for patients with LC, as the benefits include reduced fatigue, improved activity level, emotional function, abdominal symptoms, systemic symptoms, and health concerns. Similar results, but in other populations, were found by Al-Mhanna et al. (2024)^18^, who investigated the impact of combined aerobic and resistance training in patients with risk factors (type 2 diabetes, overweight, and obesity) on improving quality of life measured with the SF-36. The results indicated an increase of 0.29 points [0.03 to 0.56]; *P*=0.03. On the other hand, the study by Santos et al. (2022)^19^ demonstrated that educational programs can also contribute to improving quality of life.

Patients with LC have changes in energy metabolism, reduced muscle resistance, portal hypertension, and increased oxidative stress. Aerobic exercise improves respiratory capacity, reducing fatigue and aiding in fat oxidation, while resistance training stimulates protein synthesis and combats sarcopenia associated with LC^20^ ^,^ ^21^.

Muscular resistance, both peripheral and respiratory, is significantly reduced in these individuals, directly impacting fatigue levels. Adequate training promotes increased muscle capacity, driven by the growth in the number of mitochondria and the adaptation of muscle fibers, improving energy efficiency and resistance to fatigue, factors directly related to the quality of life of patients with LC^7^ ^,^ ^21^.

To improve the quality of life of these patients with LC, it is recommended to practice aerobic exercises on a cycle ergometer for a period of eight to 12 weeks, with a frequency of two to four times a week, an intensity of 60% to 80% of VO2peak, HRmax or HRreserve and a duration of 30 to 60 minutes. Alternatively, muscle strength training can be performed three times a week, with 30-minute sessions, or even activities of daily living exercises using elastic bands, increasing the resistance of the band as the individual is able to perform 10 repetitions of each movement pattern without significant fatigue or impairment in adequate execution, for at least 12 weeks (Table 2).

Compared to the previous meta-analysis^12^, this review proved to be more comprehensive, as it used 11 databases, assessed the risk of publication bias, included a greater number of studies and presented statistically significant and clinically relevant results.

This meta-analysis presented the following strengths: (1) all studies evaluated by RoB 2 presented low risk of bias (Figure 2); (2) low inconsistency (I2=20%; *P*=0.29; Figure 3); (3) inclusion of studies that met the eligibility criteria aligned with the objective of the meta-analysis; (4) despite the small number of studies (k=5), the total sample of 178 participants provided precision to the results of the analysis^16^ (FIGURES 3 AND 4); and 5) there was no evidence of publication bias (Figure 4). These factors contributed to the high reliability of the evidence obtained (Table 4).

Finally, it is worth highlighting that, although the methodological quality of the studies was classified as moderate by the Jadad scale, the lack of blinding was a common aspect among them (Table 3). The impossibility of blinding the participants and the professionals who applied the physical exercises may have influenced the results. However, in controlled and randomized studies involving physical exercises, total blinding is not feasible^22^. Another relevant aspect is the scarcity of studies that exclusively evaluate the impact of muscular and/or inspiratory strength training in patients with LC (Table 2), indicating the need for future research in this area.

## CONCLUSION

The results of this meta-analysis demonstrated that the practice of aerobic and/or resistance physical exercises significantly improved the quality of life of patients with CH. The increase of 0.27 points in the quality of life score reinforces the importance of physical exercises as a complementary therapeutic strategy for this population.

Therefore, it is recommended to implement supervised physical exercise programs for patients with CH, considering individualized parameters of intensity and duration. This approach can contribute to better management of the disease, providing greater well-being and functional autonomy to affected individuals.
